# Proteins of the VEGFR and EGFR pathway as predictive markers for adjuvant treatment in patients with stage II/III colorectal cancer: results of the FOGT-4 trial

**DOI:** 10.1186/s13046-014-0083-8

**Published:** 2014-10-02

**Authors:** Thomas Thomaidis, Annett Maderer, Andrea Formentini, Susanne Bauer, Mario Trautmann, Michael Schwarz, Wiebke Neumann, Jens Martin Kittner, Arno Schad, Karl-Heinrich Link, Johannes Wilhelm Rey, Arndt Weinmann, Arthur Hoffman, Peter Robert Galle, Marko Kornmann, Markus Moehler

**Affiliations:** I. Medical Department, Johannes-Gutenberg University, Mainz, Germany; Department of Surgery, University Hospital, Ulm, Germany; Department of Pathology, Johannes-Gutenberg University, Mainz, Germany; Department of Surgery, Asklepios Clinic, Wiesbaden, Germany; Medical Department, Marienhospital, Frankfurt, Germany

**Keywords:** Stage II/III colorectal cancer, Predictive biomarkers, EGFR, VEGFR, Amphiregulin, Epiregulin, PTEN, Hif-1 alpha, Adjuvant chemotherapy

## Abstract

**Background:**

Unlike metastatic colorectal cancer (CRC) there are to date few reports concerning the predictive value of molecular biomarkers on the clinical outcome in stage II/III CRC patients receiving adjuvant chemotherapy. Aim of this study was to assess the predictive value of proteins related with the EGFR- and VEGFR- signalling cascades in these patients.

**Methods:**

The patients' data examined in this study were from the collective of the 5-FU/FA versus 5-FU/FA/irinotecan phase III FOGT-4 trial. Tumor tissues were stained by immunohistochemistry for VEGF-C, VEGF-D, VEGFR-3, Hif-1 α, PTEN, AREG and EREG expression and evaluated by two independent, blinded investigators.

Survival analyses were calculated for all patients receiving adjuvant chemotherapy in relation to expression of all makers above.

**Results:**

Patients with negative AREG and EREG expression on their tumor had a significant longer DFS in comparison to AREG/EREG positive ones (p< 0.05). The benefit on DFS in AREG-/EREG- patients was even stronger in the group that received 5-FU/FA/irinotecan as adjuvant treatment (p=0.002). Patients with strong expression of PTEN profited more in terms of OS under adjuvant treatment containing irinotecan (p< 0.05). Regarding markers of the VEGFR- pathway we found no correlation of VEGF-C- and VEGFR-3 expression with clinical outcome. Patients with negative VEGF-D expression had a trend to live longer when treated with 5-FU/FA (p=0.106). Patients who were negative for Hif-1 α, were disease-free in more than 50% at the end of the study and showed significant longer DFS-rates than those positive for Hif-1 α (p=0.007). This benefit was even stronger at the group treated with 5-FU/FA/irinotecan (p=0.026). Finally, AREG-/EREG-/PTEN+ patients showed a trend to live longer under combined treatment combination.

**Conclusions:**

The addition of irinotecan to adjuvant treatment with 5-FU/FA does not provide OS or DFS benefit in patients with stage II/III CRC. Nevertheless, AREG/EREG negative, PTEN positive and Hif-1 α negative patients might profit significantly in terms of DFS from a treatment containing fluoropyrimidines and irinotecan. Our results suggest a predictive value of these biomarkers concerning adjuvant chemotherapy with 5-FU/FA +/− irinotecan in stage II/III colorectal cancer.

**Electronic supplementary material:**

The online version of this article (doi:10.1186/s13046-014-0083-8) contains supplementary material, which is available to authorized users.

## Introduction

Colorectal cancer (CRC) is the third most common malignancy and one of the most common causes of cancer-related death in the western world [1]. The progress that has been made over the past decades in the field of systemic therapy, radiological imaging and surgical interventions resulted in a significant improvement of overall survival (OS) rates especially in patients with distant metastasis [2]. Currently, the 2-year estimated OS rates in patients with metastatic disease reach 22% [2], whereas patients with local advanced colorectal cancer following adjuvant chemotherapy show a 5-year disease free survival (DFS) in 73.3% [3]. The development of new therapeutic strategies associated with improved OS and DFS rates in patients with CRC is still the focus of clinical research, but requires a detailed understanding of the biological processes that regulate the establishment and progression of malignant tumors.

One of the main difficulties to establish efficient therapies for human cancer is the great heterogeneity of the disease. Although many genetic and epigenetic alterations have been identified in cancer cells, the elucidation of their role as potential therapeutic targets remains a great challenge. In CRC two developmental pathways represent currently therapeutic objectives in clinical practice: the vascular endothelial growth factor receptor- (VEGFR-) and the epidermal growth factor receptor- (EGFR-) pathway.

VEGF-C and VEGF-D are ligands to VEGFR-2 and VEGFR-3 [4] and are associated with tumor growth and metastasis in multiple cancers [5-7]. In CRC the activation of the VEGFR-3 mediated cascade leads, in crosstalk with other intracellular pathways [8-10], to angiogenesis and mainly to lymphangiogenesis and therefore is involved in lymphatic metastasis [11-13]. In hypoxia, survival is associated with the formation of blood vessels and is being promoted via activation of VEGF. In such conditions the transcription factor hypoxia-inducible factor (Hif-1) α activates a large number of genes including VEGF via binding in its regulatory region [14]. Its role in promoting angiogenesis and invasion has been shown in multiple cancers [15-18], whereas recent studies have provided evidence of Hif-1α mediated resistance to radiochemotherapy, suggesting Hif-1 α as a putative therapeutic target [19,20].

On the other hand, the stimulation of EGFR results, through the activation of a complex network of pathways, including the MAPK kinase cascade and P13K/ATK pathway, in an increased cellular proliferation, angiogenesis and loss of apoptosis [21,22]. Amphiregulin (AREG) and epiregulin (EREG) are, among others, ligands of EGFR and activate the EGFR mediated intracellular cascade [23]. Increased concentrations of both proteins have been found in various cancers including CRC [24-26], whereas their potential therapeutic use has been suggested in vitro and in vivo [27,28]. Similarly, the tumor suppressor gene PTEN regulates negatively the P13K/ATK pathway acting as a downstream effector of EGFR [29]. Inactivation or loss of PTEN protein expression is found in up to 30% of sporadic CRC [30,31] and is likely to be associated with resistance to anti-EGFR monoclonal antibodies in KRAS-wt patients [32].

In light of the high-degree of complexity and crosstalk of the biological systems it is important to identify the predictive factors for clinical outcomes to achieve treatment optimization. In contrast to metastatic CRC, there are to date no comparative studies that focus on a correlation of VEGFR and EGFR related pathways with the clinical outcome in patients with local advanced colon cancer. The aim of this study was therefore to examine and compare the effect of combined adjuvant chemotherapy with 5-FU/FA (folinic acid) versus the combination of 5-FU/FA and irinotecan in patients with locally advanced colon cancer in relation to tumor VEGF-C, VEGF-D, VEGFR-3, Hif-1 α, EREG, AREG and PTEN expression.

## Materials and methods

### Patients

The patient data examined in this study (n = 269) originate from the collective of the 5-FU/FA/irinotecan vs. 5-FU/FA trial of the German ‘Research Group Oncology of Gastrointestinal Tumors’ (FOGT-4). Aim of the study was to investigate the efficacy and safety of adding irinotecan to 5-FU/FA to patients with locally advanced colon cancer in adjuvant setting. Primary end point was OS, and secondary end points were recurrence-free survival, toxicity, quality of life and determination of predictive and prognostic makers for treatment. Eligibility criteria were the R0-resection of a locally advanced adenocarcinoma of the colon in pathological UICC stage III (pTxpNposM0R0) or IIB (pT4pN0M0R0). Patients had to be over 18 years old and gave written informed consent according to the Helsinki protocol before entering the study, which was approved by the ethics committees of the participating institutions (Ref. Nr: #727/2001). The study was approved by the Ethics Committee of the University of Ulm.

### Treatment

The participants were randomized into two treatment arms. The 5-FU/FA/irinotecan group received infusional irinotecan 80 mg/m^2^ over 60 minutes and FA 500 mg/m^2^ over 2 hours, followed by an infusion of 5-FU 2000 mg/m^2^ over 24 hours as previously described [33]. Patients in the 5-FU/FA therapy arm received FA 200 mg/m^2^ as short intravenously (i.v) infusion combined with the i.v. administration of 5-FU 2000 mg/m^2^ over 2 hours as described before [33,34]. The duration of the adjuvant chemotherapy was ca. 6 months.

### Immunohistochemistry

The expression of AREG, EREG, VEGF-C, VEGF-D, VEGFR-3, PTEN and Hif-1 α was analyzed by immunohistochemistry (IHC). Paraffin-embedded tissue samples were obtained from 185 patients for AREG, 183 for EREG, 204 for VEGF-C, 203 for VEGF-D, 202 for VEGFR-3, 115 for Hif-1 α and 122 for PTEN due to limited availability of material (Additional file 1, Table 1).

**Table 1:** **Marker expression on tumor tissues in patients with stage II/III CRC treated with 5-FU/FA vs 5-FU/FA/irinotecan**

	**Total study population**	**5-FU/FA/irinotecan**	**5-FU/FA**
	n = 269	n = 136 (50.6%)	n = 133 (49.4%)
**AREG**	185	94	91
negative	164 (88.6%)	84 (89.4%)	80 (87.9%)
positive	21 (11.4%)	10 (10.6%)	11 (12.1%)
**EREG**	183	90	93
negative	143 (78.1%)	69 (76.7%)	74 (79.6%)
positive	40 (21.9%)	21 (23.3%)	19 (20.4%)
**VEGF-C**	204	106	98
negative	91 (44.6%)	51 (48.1%)	40 (40.8%)
positive	113 (55.4%)	55 (51.9%)	58 (59.2%)
**VEGF-D**	203	106	97
negative	61 (30%)	35 (33%)	26 (26.8%)
positive	142 (70%)	71 (77%)	71 (73.2%)
**VEGFR-3**	202	103	99
negative	115 (56.9%)	57 (55.3%)	58 (58.6%)
positive	87 (43.1%)	46 (44.7%)	41 (41.4%)
**Hif-1 alpha**	115	62	53
negative	91 (79.1%)	49 (79%)	42 (79.2%)
positive	24 (20.9%)	13 (21%)	11 (20.8%)
**PTEN**	122	64	58
negative	74 (60.7%)	38 (59.4%)	36 (62.1%)
positive	48 (39.3%)	26 (40.6%)	22 (37.9%)

Three μm thick tissue sections were cut and mounted on super frost slides. These were deparaffinized, rehydrated and peroxidase blocked (3% H_2_O_2_ in methanol, 30 min). After blocking of nonspecific protein binding sites by using different strategies (Table 2), slides were incubated with the respective primary antibodies AREG and EREG (AF262 and AF1195, both R&D Systems), VEGF-C, VEGF-D, VEGFR-3 and Hif-1α (sc-9047, sc-13085, sc 321, sc-53546, all Santa Cruz Biotechnology,) and PTEN (9188 s, Cell Signaling; more details in Table 2). After incubation with secondary antibody the specific antibody binding was visualized using DAB solution (Dako, Germany). The tissues were counterstained by hemalaun solution (Dako, Germany). Between each step of staining the specimens were washed in DPBS.

**Table 2:** **Methods for the detection of immunohistochemical expression of the tested markers**

	**Company**	**Antigen**	**Blocking**	**1.AK**	**2.AK**
		**retrieval**	**30′RT**		
AREG	R&D Systems	0,1 M	5% swine	1:75,	LSAB + System-HRP
	AF262	EDTA buffer pH8	serum	over night	K 0690
EREG	R&D Systems	-	5% swine	1:100	LSAB + System-HRP
	AF1195		serum	over night	K 0690
VEGF-C	Sant Cruz	-	FFP	1:50	Dako Real™EnVision™Detection System
	sc-9047			over night	K 5007
VEGF-D	Sant Cruz	0.01 M	FFP	1:50	Dako Real™EnVision™Detection System
	sc-13085	Citrat buffer		2 h RT	K 5007
VEGFR-3	Sant Cruz	0.01 M	FFP	1:100	LSAB + System-HRP
	sc-321	Citrat buffer		2 h RT	K 0690
Hif-1 α	Sant Cruz	0,1 M	5% swine	1:50	LSAB + System-HRP
	sc-53546	EDTA buffer pH8	serum	over night	K 0690
PTEN	Cell Signaling	0.01 M	5% goat	1:75	Dako Real™EnVision™Detection System
	9188 s	Citrat buffer	serum	over night	K 5007

*RT* room temperature, *FFP* fresh frozen plasma.

Evaluation of staining was performed by two independent, blinded investigators.

### Statistical analysis

The staining was evaluated semiquantitatively per intensity and the extent of the stained tumor area. A cut-off of 25% stained tumor cells were considered as positive staining for the markers. The survival analysis was performed by using the Kaplan-Meier method and the log rank test. To investigate the association between the results of immunohistochemistry obtained for all markers and clinical-pathological parameters, univariate statistical analysis were performed using Pearson’s Chi-2 test or Fisher’s exact test.

## Results

### Immunohistochemical analysis in respect to groups of treatment

**Figure 1:**
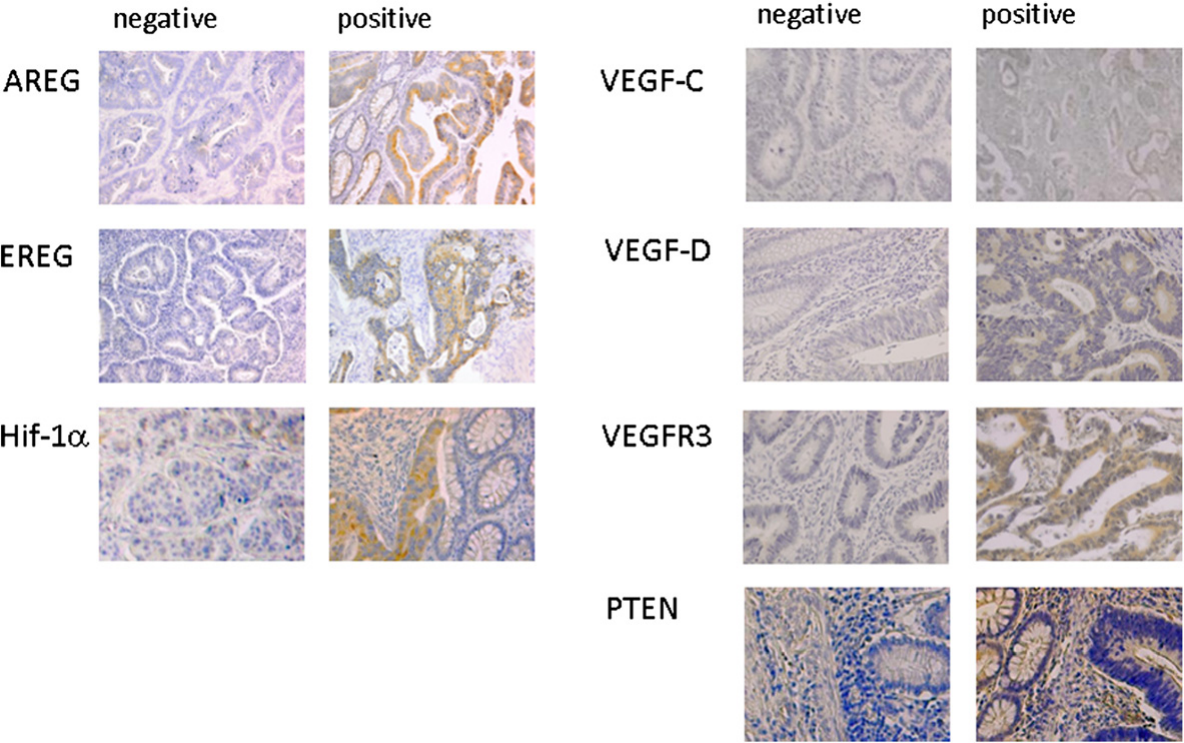
**Immunochistochemistry for AREG, EREG, Hif-1 α, VEGF-C, VEGF-D, VEGFR-3 and PTEN in tumour tissues obtained from patients with stage II/III CRC.** According to the intensity and extend the stained tumor area samples were classified as positive or negative.

As seen in Table 1 the number of tissue samples that were stained for all markers varied between 122 -for PTEN- and 204 -for VEGF-C. Nevertheless, there was an equal distribution of the patients in regards of treatment and positivity of markers. In the statistical analysis of EGFR pathway 11.4%, 21.9% and 39.3% of the samples were found positive for AREG-, EREG- and PTEN- expression respectively. Regarding the VEGFR pathway 55.4%, 70% and 43.1% of the specimens showed positivity for VEGF-C, VEGF-D and VEGFR-3. The analysis of Hif-1 α included a total of 115 tissue samples. A positive staining for Hif-1 α was observed in 24 cases (20.9%). In Figure 1 examples of positive and negative immunohistochemical staining are shown for each marker.

### General statistical analysis

In terms of efficacy, we detected no difference regarding DFS and OS after the addition of irinotecan to 5-FU/FA. Furthermore, there was no correlation between age, UICC stage, T-, N- stage and expression of the tested markers (data not shown).

### Results for the EGFR- pathway

**Figure 2:**
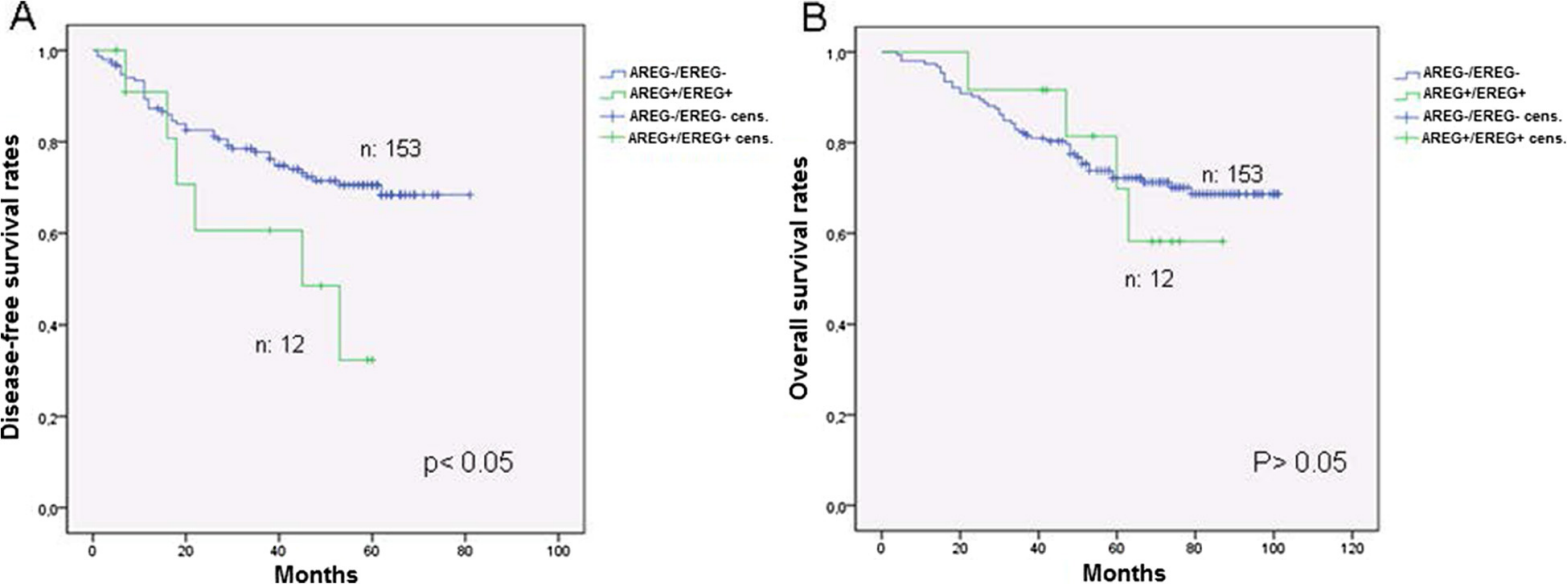
**Survival analysis in patients receiving adjuvant treatment in relation to AREG- and EREG- expression. A**. Disease-free survival rates. **B**. overall survival rates.

**Figure 3:**
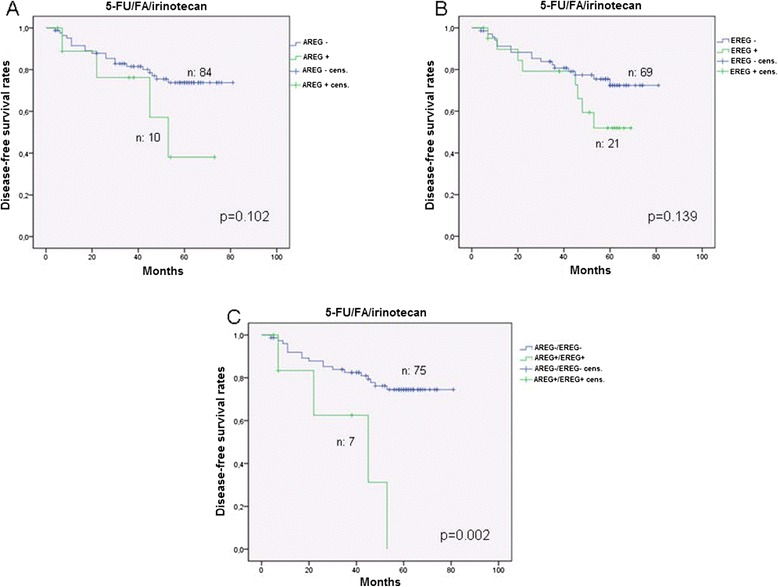
**Survival analysis in patients under adjuvant 5-FU/FA/irinotecan in relation to AREG/EREG- expression. A**. Disease-free survival in relation to AREG expression on tumor tissues. **B**. Disease-free survival in relation to EREG expression on tumor tissues. **C**. Disease-free survival in relation to AREG and EREG expression on tumor tissues.

**Figure 4:**
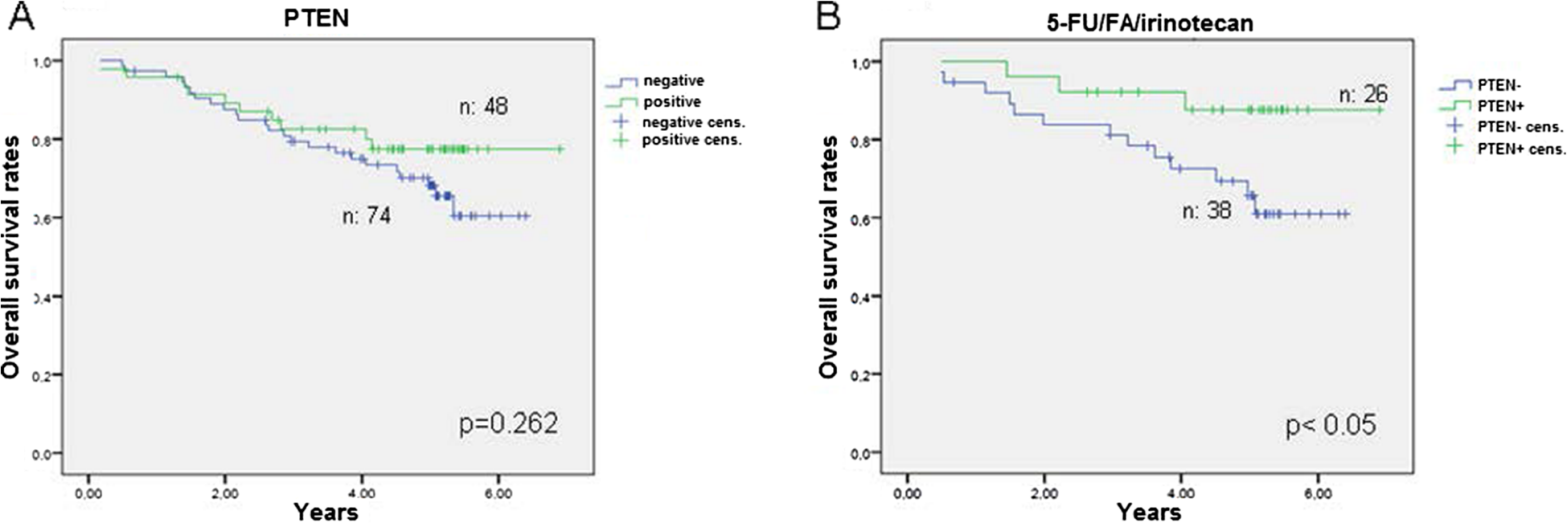
**Overall survival analysis for patients according to tumor PTEN-status. A**. in the whole population **B**. in the subgroup treated with adjuvant 5-FU/FA/irinotecan.

We found no statistically significant difference in respect to the expression status of AREG or EREG and the survival of the patients in the total study population. However, patients with negative AREG and EREG expression had a significant longer DFS in comparison to AREG and EREG positive ones independent of the adjuvant treatment (Figure 2A, p < 0.05). The OS though, did not differ significantly (Figure 2B). Patients with a negative AREG- or EREG- state showed a trend for a longer DFS over a period of about 7 years under the combined combination of 5-FU/FA/irinotecan (Figures 3A and 3B). Patients with a negative state for AREG and EREG benefited strongly from the addition of irinotecan in the adjuvant backbone treatment, in terms of DFS over 80 months (Figure 3C, p = 0.002). The median DFS for AREG- and EREG- positive patients was 45 months under 5-FU/FA/irinotecan, whereas more than half of the patients with negative immunohistochemistry for AREG and EREG were disease free at the end of the study. Furthermore, PTEN expression appeared to have no significant impact on the survival rates of the total study population (Figure 4A). However, in the group of 5-FU/FA/irinotecan patients positive for PTEN benefited in terms of OS compared to PTEN- negative ones (p < 0.05, Figure 4B).

### Results for the VEGFR- pathway

**Figure 5:**
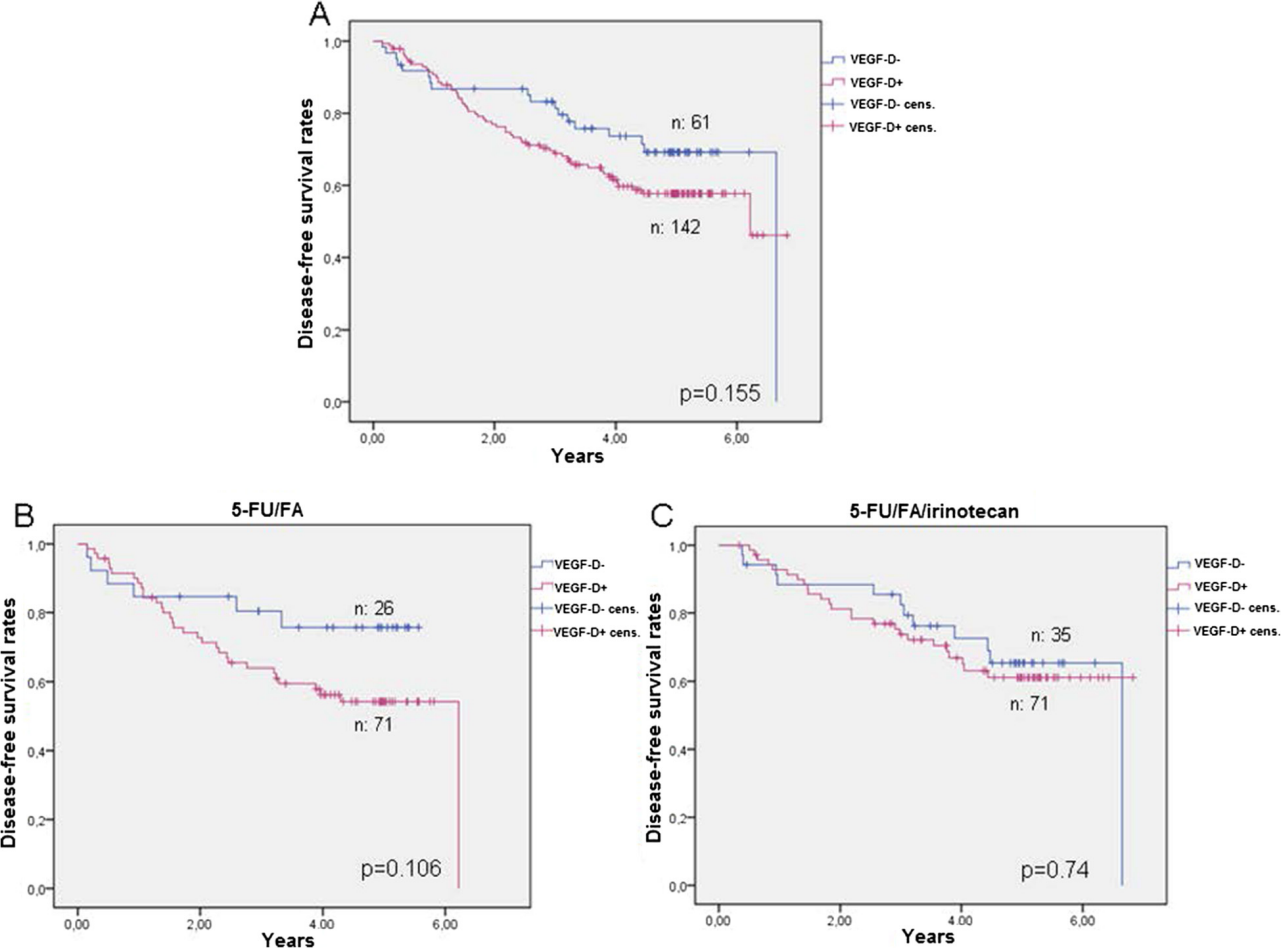
**Disease-free survival in patients under adjuvant 5-FU/FA versus 5-FU/FA/irinotecan in relation to VEGF-D expression. A**. Disease-free survival rates in the total population, **B**. Patients under adjuvant 5-FU/FA, **C**. Patients under adjuvant 5-FU/FA/irinotecan.

The expression of VEGF-D, VEGF-C and VEGFR-3 in tumor tissues did not show a direct impact on survival in the total study population. In the 7-year survival, a trend for a longer DFS was found for patients with no VEGF-D expression (p = 0.155, Figure 5A). While VEGF-D negative patients showed a trend to remain disease free towards treatment with 5-FU/FA (p = 0.106, Figure 5B), no benefit in survival was observed under the combined adjuvant treatment (Figure 5C).

**Figure 6:**
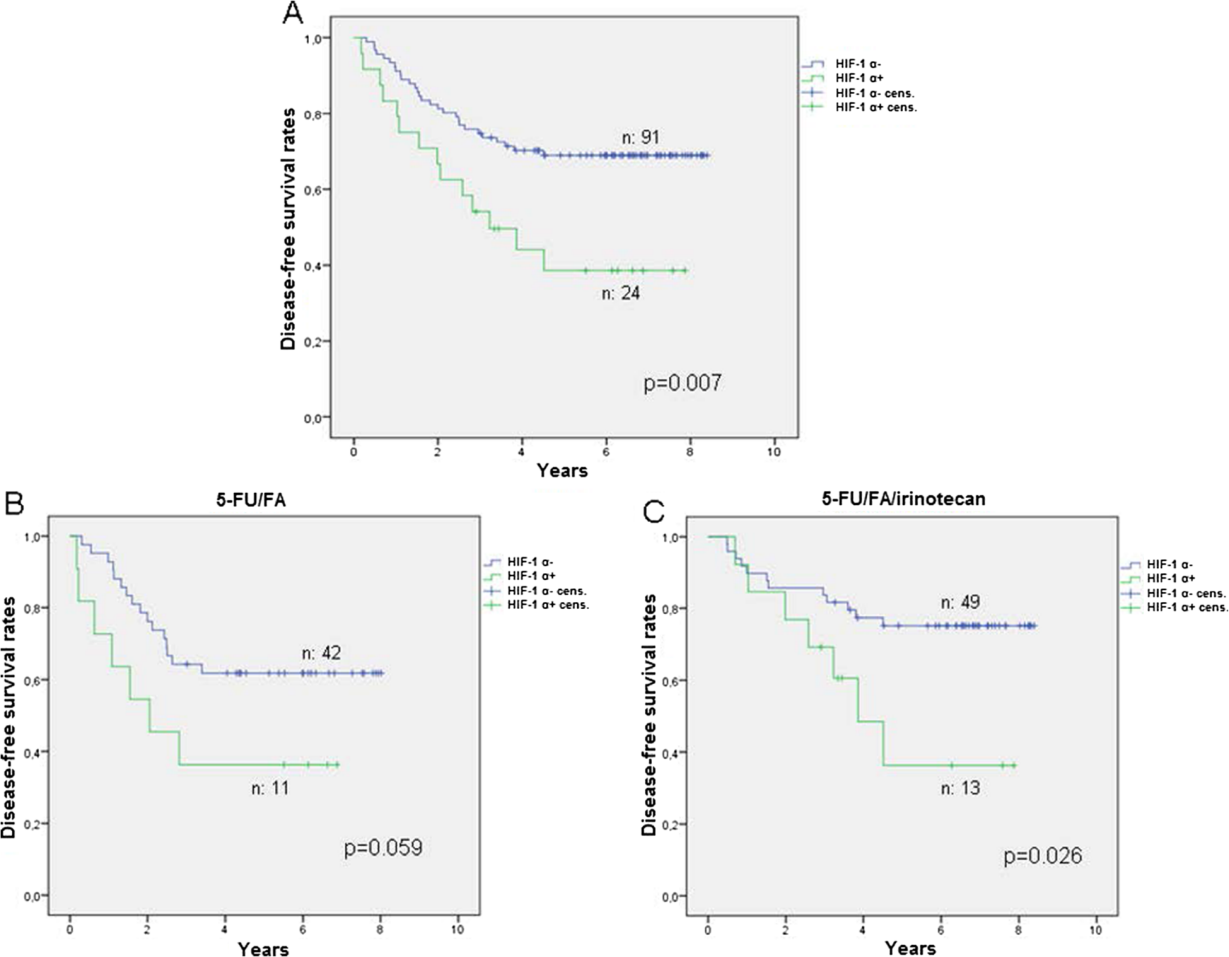
**Disease-free survival in patients under adjuvant 5-FU/FA versus 5-FU/FA/irinotecan in relation to Hif-1 α expression. A**. Disease-free survival rates in the total population, **B**. Patients under adjuvant 5-FU/FA, **C**. Patients under adjuvant 5-FU/FA/irinotecan.

In respect to Hif-1 α, the median DFS of patients with positive status was 3.3 years for the entire group, 2.1 years for the 5-FU/FA group and 3.9 years for the 5-FU/FA/irinotecan group. Patients who were negative for Hif-1 α, were in more than 50% disease-free at the end of the examination period in all above cases (p = 0.007, p = 0.059 and p = 0.026 respectively, Figure 6).

### Combining results for both pathways

**Figure 7:**
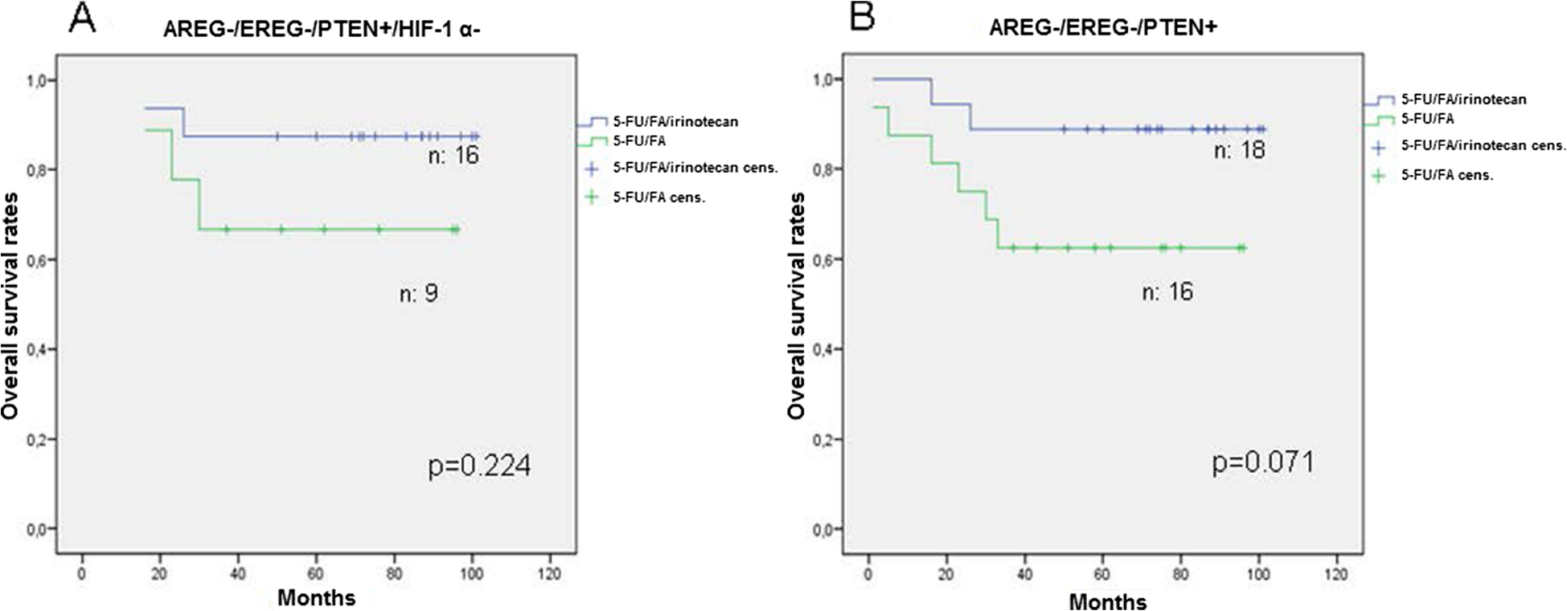
**Survival analysis in relation to collective biomarker expression.** Overall survival in patients treated with adjuvant 5-FU/FA versus 5-FU/FA/irinotecan in patients negative for AREG, EREG, Hif-1 α and positive for PTEN **(A)** and in patients negative for AREG, EREG and positive for PTEN **(B)**.

Patients who had a negative expression for AREG, EREG, Hif-1 α and positive for PTEN represented 9% of the total study population and showed not significant prolonged OS and DFS under 5-FU/FA/irinotecan treatment (Figure 7A). 34 patients were AREG-/EREG-/PTEN + and had a trend to live longer under the triple combination (p = 0.071, Figure 7B).

## Discussion

There is an increasing number of reports concerning the role of biomarkers on the identification of those patients who will benefit from treatment with targeted agents [35-38]. In this study we evaluated the impact of the tyrosine kinase receptor ligands VEGF-C, -D, AREG, EREG, as well as Hif-1 α, PTEN and of the VEGFR-3 on disease recurrence and survival in patients with CRC receiving adjuvant chemotherapy. This is –to our knowledge- the first study analyzing all these parameters in the specific target population of CRC stage II/III.

Indeed, unlike metastatic CRC, there have been only a limited number of trials that attempted to determine prognostic and predictive biomarkers in resected CRC. Current evidence suggests that the presence of high microsatellite instability (MSI-h) in tumor tissues is associated with a longer DFS and OS in non-metastatic CRC [33,39-41]. The ongoing B-CAST trial [42] evaluates the protein expression of thymidine phosphorylase (TP), dihydropyrimidine dehydrogenase (DPD), EGFR and VEGF in 2128 patients with stage III CRC, but the results are yet to be published. Other studies proposed loss of SMAD4 [43], high expression of wt-p53 [44], NF-kB negativity or JNK positivity [45] as possible biomarkers associated with longer time to relapse in stage II/III CRC. Nevertheless, there has been so far no sufficient validation of their use in clinical practice.

Similarly to the findings of Tikidzhieva et al. [33] we observed in our analysis no benefit of the addition of irinotecan to adjuvant 5-FU/FA in terms of DFS and OS (data not shown). These results are consistent with those of previously published phase III trials [46-48]. Nevertheless, we cannot exclude the possibility that irinotecan might be effective for subgroups of patients with stage II/III CRC whose tumors present specific molecular patterns.

As depicted in Table 1 the tumor tissues in our study showed higher levels of VEGF-C, VEGF-D, VEGFR-3 expression when compared to proteins related to the EGFR pathway. Nevertheless, these expression levels should not be considered representative since they may vary among tumors with different entity and TNM stage. It is characteristic, that the immunohistochemical detection of VEGF-C and VEGF-D in stage II/III colorectal adenocarcinoma varies between 43%-77% and 50%-64% [49,50] respectively.

In our study we demonstrated a statistical significant benefit in terms of DFS for patients who lacked AREG and EREG expression. This benefit was even stronger when AREG-/EREG- patients received adjuvant therapy including irinotecan (Figure 3). Our findings are consistent with those of Jacobs et al. who postulated that expression of AREG/EREG can predict the outcome for KRAS wt stage IV CRC when treated with cetuximab and irinotecan [51]. In this study a positive prognosis associated with AREG/EREG expression for EGFR targeted treatment was shown. This discrepancy in comparison to our results might be attributed to the applied treatment with anti-EGFR agents or to the metastasized stage of the examined patients in Jacobs’ study. Indeed, EGFR blockage for stage II/III CRC patients under adjuvant treatment has not been proven beneficial so far [52]. Furthermore, the RAS-status was not tested in our study since the limited number of samples in the tested subgroups might lead to false interpretations.

Emerging data from in vitro studies with gastric [53] and colorectal [54] cancer cell lines demonstrated a synergistic effect of EGFR-inhibitors and irinotecan. Yashiro et al. showed that EGFR-inhibition could enhance the activity of SN-38, an active metabolite of irinotecan, in SN-38 resistant gastric cancer cell lines [53]. They proposed a down-regulation of SN-38 metabolism related genes through EGFR-inhibition, which might also explain the longer DFS of AREG and EREG negative patients under irinotecan treatment in our study.

Enhancement of SN-38 efficacy has also been demonstrated in ovarian cancer cells that had high PTEN expression [55]. Herein the proposed mechanism was a synergistic inhibition of topoisomerase-I activity. In deed, in our study PTEN + patients treated with 5-FU/FA/irinotecan had significant longer OS than PTEN- patients, which has also been shown from our group in advanced gastric cancer before [56].

In the last part of our study we examined whether proteins of the VEGFR- pathway could play a predictive role to adjuvant chemotherapy in patients with CRC UICC II and III. We noticed a trend for a longer DFS in VEGF-D negative patients under the treatment with 5-FU/FA (Figure 5B). A further relation of VEGF-C or VEGF-R3 with the survival of patients was not observed. These results are consistent with the findings of the AVANT and NSABP C08 trials published previously [57,58]. Both of these trials reported no benefit from the addition of bevacizumab to the combination of fluoropyrimidines and oxaliplatin in the adjuvant treatment of stage II/III CRC. Regarding Hif-1 α expression on tumor tissues we demonstrated a statistically significant prolonged DFS for patients with negative Hif-1 α expression, especially for those under irinotecan treatment. In agreement with previous in vitro and in vivo models [59] we showed that the anti-tumor efficacy of irinotecan is stronger when Hif-1 α on tumor tissues is down regulated. Since irinotecan inhibits the accumulation of Hif-1 α [60,61], our findings suggest a further mechanism of interaction between cytotoxicity of irinotecan and Hif-1 α.

In the subgroup of AREG-/EREG-/Hif-1 α-/PTEN + patients we found a non statistically significant survival benefit under the combined treatment combination. The lack of significance could be attributed to the small number of patients with the specific biomarker combination. However, AREG-/EREG-/PTEN + patients showed a clear trend to profit from the addition of irinotean to the adjuvant regime (Figure 7).

## Conclusions

There is an increasing interest in personalized therapy for colon cancer patients receiving adjuvant therapy. This is -to our knowledge- the first study investigating the role of multiple biomarkers of the EGFR- and VEGFR- pathway on the treatment outcome in patients with stage II/III CRC. We showed that an adjuvant therapy containing irinotecan might be beneficial for AREG/EREG negative, PTEN positive and Hif-1 α negative patients. However, the decision of the adjuvant regime should be always based upon the clinical characteristics of the patients including age, pre-existing treatment and side effects (i.e. oxaliplatin induced neuropathy). Nevertheless, further prospective studies including a large number of patients with stage II/III CRC are necessary in order to evaluate which molecular patterns might serve as predictive markers for treatment outcome in these patients.

## Additional file

Additional file 1:
**Consort Diagram of the study process.**

## Abbreviations

VEGFR-3: Vascular endothelial growth factor receptor 3
EGFR: Epidermal growth factor receptor
VEGF: Vascular endothelial growth factor
AREG: Amphiregulin
EREG: Epiregulin
Hif-1 α: Hypoxia-inducible factor 1 alpha
FOGT: Research group oncology of gastrointestinal tumors
OS: Overall survival
DFS: Disease free survival
CRC: Colorectal cancer
FA: Folinic acid
IHC: Immunohistochemistry
MSI-h: High microsatellite instability
TP: Thymidine phosphorylase
DPD: Dihydropyrimidine dehydrogenase
