# Supplementary material for: Proteins of the VEGFR and EGFR pathway as predictive markers for adjuvant treatment in patients with stage II/III colorectal cancer: results of the FOGT-4 trial
Source: J Exp Clin Cancer Res. 2014 Oct 2;33(1):83. doi: 10.1186/s13046-014-0083-8 (PMC4192339; doi:10.1186/s13046-014-0083-8)
Supplement: Additional file 1: — Consort Diagram of the study process. [file 13046_2014_83_MOESM1_ESM.ppt]

## Slide 1
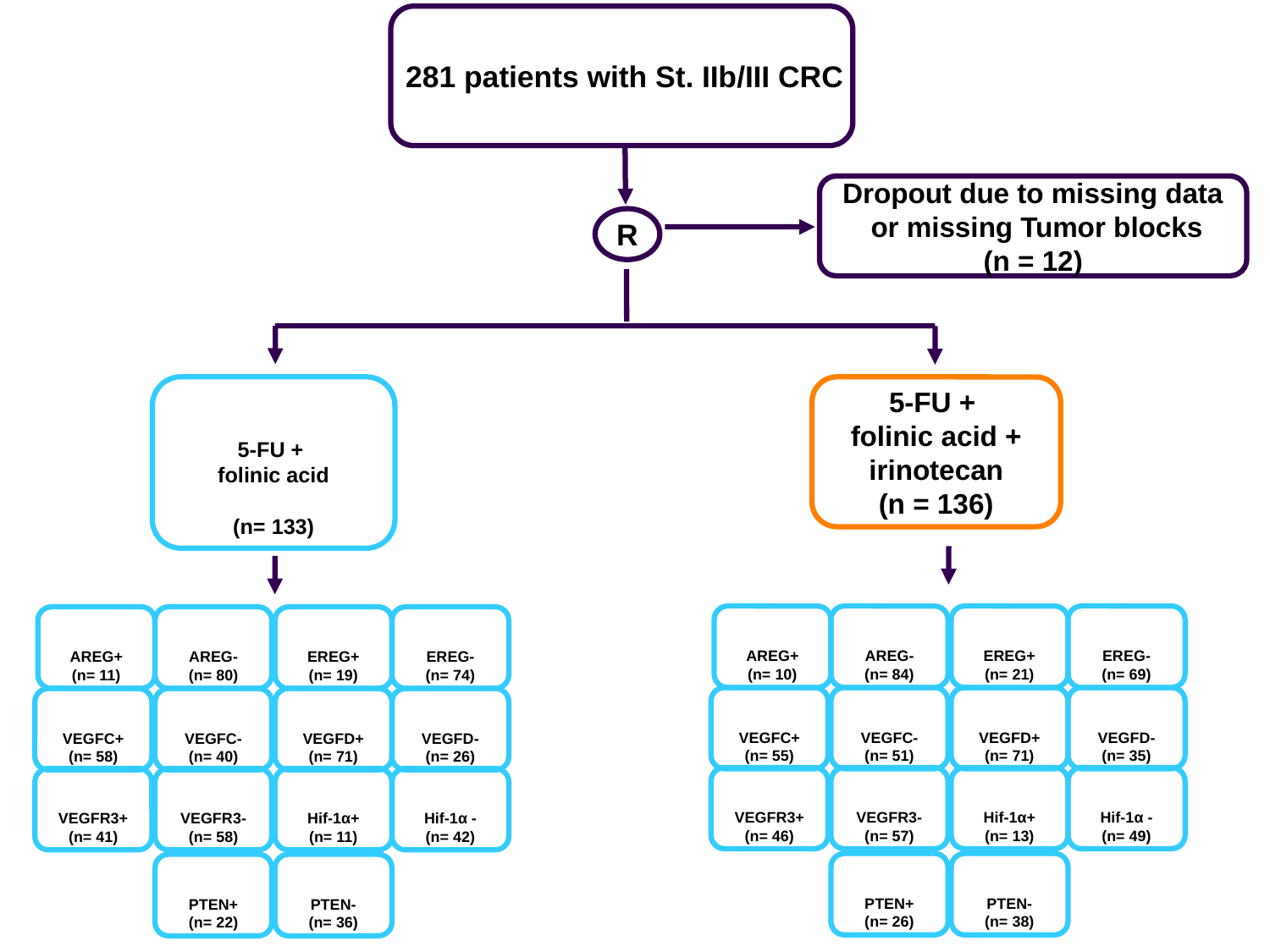

281 patients with St. IIb/III CRC
Dropout due to missing data
 or missing Tumor blocks
(n = 12)
R
5-FU +
folinic acid
(n= 133)
5-FU +
folinic acid + irinotecan
(n = 136)
AREG+
(n= 10)
AREG-
(n= 84)
EREG+
(n= 21)
EREG-
(n= 69)
VEGFC+
(n= 55)
VEGFC-
(n= 51)
VEGFD+
(n= 71)
VEGFD-
(n= 35)
VEGFR3+
(n= 46)
VEGFR3-
(n= 57)
Hif-1α+
(n= 13)
Hif-1α -
(n= 49)
PTEN+
(n= 26)
PTEN-
(n= 38)
AREG+
(n= 11)
AREG-
(n= 80)
EREG+
(n= 19)
EREG-
(n= 74)
VEGFC+
(n= 58)
VEGFC-
(n= 40)
VEGFD+
(n= 71)
VEGFD-
(n= 26)
VEGFR3+
(n= 41)
VEGFR3-
(n= 58)
Hif-1α+
(n= 11)
Hif-1α -
(n= 42)
PTEN+
(n= 22)
PTEN-
(n= 36)
